# Zoonotic Disease: Knowledge, Attitude and Practice of Dairy Farm Owner in Wolaita Sodo District, Ethiopia

**DOI:** 10.1002/vms3.70197

**Published:** 2025-01-15

**Authors:** Getachew Derbew Belay, Amare Bihon Asfaw, Hagazi Fantay Tadesse, Asma Seid

**Affiliations:** ^1^ College of Veterinary Medicine and Animal Science Samara University Samara Ethiopia; ^2^ School of Veterinary Medicine Woldia University Woldia Ethiopia; ^3^ College of Veterinary Science Mekelle University Mekelle Ethiopia

**Keywords:** attitude, dairy farm, knowledge, practice, Sodo, zoonoses

## Abstract

**Background:**

Lack of knowledge regarding zoonotic transmission, prevention and control measures is a potential high risk for the occurrence of zoonotic diseases.

**Objective:**

The study aimed to assess knowledge, attitude and practices of dairy farm participants concerning zoonoses.

**Animals:**

A cross‐sectional study was conducted from March to August 2022 in and around Sodo town, using a questionnaire among dairy farm participants (*n* = 123). The structured questionnaire consisted of 48 items to evaluate knowledge (12), attitude (10) and practices (6) related to zoonotic disease–risks from livestock products and birth products was administered to the systematically and randomly selected respondents. Linear regression analyses were used to assess relations between the explanatory variables and the three indexes.

**Results:**

The overall positive response for knowledge, attitude and practice subscores were 65%, 74% and 59% respectively. 93% of participants know that disease can transmit from animal to human. Besides, eating uncooked meat (92%), drinking raw milk (85%) and collecting aborted foetuses and placenta with bare hand (80%) are known sources of infection. Participants who thought zoonotic diseases can be treated, controlled and prevented were 78%, 80% and 81%, respectively. Among participants, 74% showed positive attitude towards risk of acquiring disease through the consumption of raw meat and milk. The participants who believed that apparently healthy animals can be a source of infection were 42%. Education level was positively associated with better knowledge, attitude and practice towards zoonoses. Furthermore, farm type, size and respondent's residence were also associated with better practice of zoonotic disease prevention (*p* < 0.05).

**Conclusion:**

Despite good knowledge, attitude and practice about zoonotic diseases' control and prevention, there are some serious knowledge and practice shortcomings. Awareness creation and training programs for the members of the dairy producers on zoonotic diseases and their transmission mechanism might help the effort in public health significance of zoonotic disease prevention.

## Graphical abstract

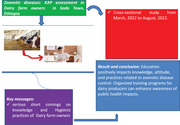

## 1 Introduction

Livestock production is important in Ethiopia's agricultural economy (Wodajoa et al. 2020). It is contributing a great role in the livelihood of the rural community, particularly, dairy cattle production has a paramount role in Ethiopia's economy where livestock and its products are important source of food and income generation (Management Entity 2021; Sintayehu et al. 2008). In 2017, the sector contributed up to 40% of agricultural Gross Domestic Product (GDP), nearly 20% of total GDP, and 20% of national foreign exchange earnings (World Bank 2017). Nevertheless, livestock production can also be a source of infection for humans, through direct contact with animals or unsafe using of their product (Klous et al. 2016), and become a source of zoonotic pathogens (World Bank 2017).

Zoonotic diseases are infectious diseases that have great public health concerns and are transmitted from animals to humans or from humans to vertebrate animals (Rahman et al. 2020; World Bank 2017). It can be transmitted to humans directly or indirectly from animals (Mortimer 2019), either by the consumption of contaminated food and water, and exposure to the pathogen during preparation and processing, or by direct contact with infected animals or humans (Smolinksi et al. 2003). According to World Economic Forum reports in 2022, it represents a growing threat to public health—about 60% of known infectious diseases and up to 75% of new or emerging infectious diseases are zoonotic in origin (Stefan 2022). Zoonoses are categorized based on their disease‐causing agents, such as bacterial, viral, parasitic, or mycotic/fungal; their reservoir hosts, either animal or human; or the life cycle of disease‐causing agents (Chomel 2009).

Developing countries including Ethiopia have a higher incidence and prevalence of zoonoses, and this is attributed to the lack of adequate control mechanism, lack of adequate infrastructure and lack of adequate information on their significance and distribution (Singh et al. 2020). In Ethiopia, 80% of households have direct relations with domestic animals, which favour an opportunity for infection and spread of disease (Pieracci et al. 2016); also, Ethiopia ranks very high in the health burden of zoonotic diseases due to having a large population of poor livestock keepers (Leta and Mesele 2014). In Ethiopia, the majority of dairy farms are small‐scale subsistence operations, with a limited number of small‐ and medium‐sized commercial dairy farms. Increased human–animal contact or interaction resulting from changes in human and animal behaviour, pathogen adaptability, change in farm practices, livestock production systems and food safety are among the triggering factors for the emergence of zoonotic diseases (Lindahl and Grace 2015).

Knowledge, attitude and practice (KAP) research is widely used in public health and conservation scholarship to collect information about public understanding of a phenomenon (knowledge), evaluative responses to a situation (attitudes) and observed actions or behaviours (practices) among a target population (Gumucio et al. 2011; WHO 2010). Zoonotic disease is a great public health concern and a direct human health hazard that may even lead to death (Rahman et al. 2020) and that may have caused an estimated 2.4 billion cases of illness and 2.7 million deaths in humans per year. In addition to its negative effect on human health, most of it affects animal health and decreases livestock production (Grace et al. 2012).

These impacts of zoonotic disease are also common in Ethiopia in general and in the study area in particular. The prevention and control of zoonotic diseases include an understanding of the factors affecting the probability that zoonoses will emerge, the likely pattern of their spread reducing risky human–animal interactions, improving the welfare of domestic and wild animals, and refining global surveillance systems for people and animals (Castillo‐Chavez et al. 2015). Currently, there are inadequate data on the KAP of Wolaita Sodo community towards zoonotic diseases. So, to overcome the problem and assess the knowledge, attitude and hygienic practice of zoonotic disease in the area, the current study needed to conduct and address the problems regarding zoonotic diseases. Therefore, the objectives of this study were to assess the knowledge and attitude of the community regarding zoonotic disease and to evaluate the community practice related to zoonotic disease in dairy farms in and around Sodo town.

## 2 Materials and Methods

### 2.1 Study Area

**FIGURE 1:**
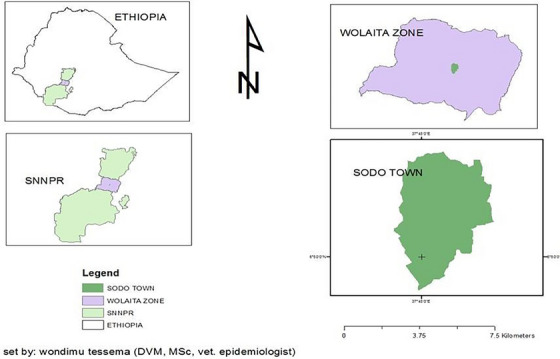
Map of the study area.

The present survey was carried out from March to August 2022 in and around Sodo town in the Wolaita Zone (Figure 1). The study site is located 390 km south of Addis Ababa and is found at 6°54′N latitude and 37°45′E longitude with an elevation between 1650 and 2980 m above sea level. The district is bounded by the Damot Gale district to the north, Humbo district to the south, Damote Woyde district to the east and Damote Sore district to the west; the annual rainfall and temperature of the area are 1000–1200 mm and 26°C–35°C, respectively. The site is classified under a mid‐altitude (‘Woyina dega’ in the local Amharic language) agroecological environment. The dry season lasts from September to February, and the rainy season remains from March to August. The livestock population of the region was estimated to be 1,097,710 cattle, 150,383 sheep, 185,250 goats, 60,055 equines and 734,924 poultry (SZOA 2021). These large livestock populations (Urban dairy farming) initiate researchers to assess the KAP of dairy farm owners towards zoonotic disease.

### 2.2 Study Population, Design and Sampling Technique

The study population was the farmers belonging to Woliata Sodo town. The target population comprised of dairy farm owners residing in and around Sodo town. Cross‐sectional questionnaire–based study design and random sampling technique were employed to select households for this study. A list of households owning dairy farms was obtained from the Sodo Town Agricultural Office, Livestock and Fishery Sector. A random sampling technique was used to select the households for the purpose of this study and a random survey of 123 urban and per‐urban dairy farmers who were actively involved in dairy production was conducted.

### 2.3 Sample Size Determination

The sample size for collecting the questionnaire data was determined by using the formula as indicated by Bonett (2002) and Arifin (2018). The required sample size was calculated using an expected Cronbach's α of 0.7 with a significance level = 0.05; confidence interval of 95%; 17 response items (for knowledge subscale); and an expected dropout rate (incomplete information rate) of 20%. A total of 123 dairy farmers were used during the survey.

### 2.4 Data Collection Method

Dairy farmers were visited and the questionnaires were administered to systematically and randomly selected sample of the population in the study area. A close‐ended questionnaire was developed and pretested to assess KAP towards zoonotic disease, a questionnaire was developed to measure participants’ knowledge about zoonotic disease, attitudes related to zoonotic disease risk and practices used to prevent zoonotic disease risks from livestock products and birth products. The demographic and farm characteristics questionnaire included information on gender, age, education, primary livelihood activity, respondent's role, residential area, farm size, farm type and others.

A questionnaire consisting of 4 sections and 32 questions with several subquestions was developed. The questionnaire contained binary, multiple selection, open‐ended and Likert‐scale questions. Several of these questions were utilized in the construction of three index variables, namely knowledge, attitude and practice scores. The final version of the KAP tool comprised 28 items in three subscales (Table 1); 12 items measuring zoonotic disease knowledge, 10 items measuring zoonotic disease risk attitude, and 6 items measuring zoonotic disease prevention practices. Knowledge subscale items had dichotomous responses (correct or incorrect) and items that were initially open‐ended questions were restructured into ‘correctly named’ versus ‘not named correctly’. Items related to attitude had dichotomous (‘agree’ vs. ‘disagree’). All items in the practice subscale had dichotomous (‘success’ vs. ‘failure’).

**TABLE 1:** KAP items’ description.

Item No.	Knowledge subscale contents	Response
K1	Knowing about zoonotic disease	Correct/incorrect
K2	Training about zoonotic disease	Correct/incorrect
K3	Eating uncooked meat can transmit diseases from animals to you	Correct/incorrect
K4	Drinking of raw milk can transmit diseases from animals to you	Correct/incorrect
K5	Get infection from environment contaminated from secretions of sick animal	Correct/incorrect
K6	Disposing aborted foetuses into the environment can spread the diseases	Correct/incorrect
K7	Contact during animal abortion can cause a public health problem	Correct/incorrect
K8	Collecting aborted foetuses and placenta with bare hand exposes you to diseases	Correct/incorrect
K9	Assisting animals during parturition with bare hand exposes you to diseases	Correct/incorrect
K10	Family or farm member sick with disease of animal origin	Correct/incorrect
K11	Transmission of diseases through milk or meat	Correct/incorrect
K12	Mention correctly three diseases that transmitted from animals to humans	Correct/incorrect
	***Attitude subscale contents***	
A1	Assisting animals in delivery with bare hand exposes you to disease risks	Agree/Disagree
A2	Collecting aborted foetuses and placenta with bare hand exposes you to disease risks	Agree/Disagree
A3	Animal health care providers can handle zoonotic disease outbreaks very well	Agree/Disagree
A4	Animal diseases are dangerous for people	Agree/Disagree
A5	Zoonotic diseases can be treated	Agree/Disagree
A6	Zoonotic diseases can be prevented	Agree/Disagree
A7	Zoonotic diseases can be controlled	Agree/Disagree
A8	Meat and milk borne diseases are fatal	Agree/Disagree
A9	No risk of disease from drinking raw milk and eating raw meat	Agree/Disagree
A10	Only sick cattle could be source of milk and meat borne disease	Agree/Disagree
	***Practice subscale contents***	
P1	Drinking of milk after boiling (pasteurization)	Success/Failure
P2	Eating of meat after cooking	Success/Failure
P3	Separating house of animals	Success/Failure
P4	Assist animal delivery with protected hands	Success/Failure
P5	Wash hands with soap after assisting animal delivery	Success/Failure
P6	Avoid any contact with aborted material	Success/Failure

### 2.5 Data Quality Assurance

Pretest was performed before starting of the full study in similar participants to see the applicability and quality of the questionnaire. Each questionnaire was checked for missed value, incompletes and consistency.

### 2.6 Data Analysis

All the collected data were coded and entered into Microsoft Excel. The questionnaires were checked for completeness before entering the data into SPSS Statistics for Windows Version 20.0 statistical software (released 2011). Descriptive statistics such as frequencies, distribution and percentages are used to summarize the data. The association of demographic characteristics of the respondents and KAP were analysed using general linear models. A KAP was prepared adding up farmer's specific questions and subquestions. A score 1.0 was awarded if the participant could choose the correct, agree and successful answers and no score granted for an incorrect, disagree and failure replies. The internal consistency of the subscales was assessed by Cronbach's α coefficient, where a Cronbach's α ≥ 0.7 was considered as acceptable. A value of Cronbach's α > 0.8 was an indicator of good reliability and Cronbach's α between 0.7 and 0.8 indicated adequate reliability. Subscales with Cronbach's α values below 0.5 indicated unacceptable internal consistency. Pearson's correlation coefficient was used to measure the relationship between subscales. Coefficient values between 0.8 and 1.0 indicated a very strong relationship, between 0.6 and 0.8 indicated a strong relationship, between 0.4 and 0.6 indicated a moderate relationship, between 0.2 and 0.4 indicated a weak relationship and a value between 0 and 0.2 indicated a very weak to no relationship.

## 3 Results

### 3.1 Socio‐Demographic Characteristics

A total of 123 dairy farmers were interviewed during the study period. Tables 2 and 3 presented the detailed demographic and farm characteristics of the study participants. More than half of the participants were farm owners (66 %) and women (58%). Less than 20% of the participants were illiterate and 56% of the participants’ dairy farming is used as their primary livelihood activity. Regarding farm characteristics, about 70.73%, 61% and 63% of the farms were urban in residence, smallholder in farm size and intensive in farm type, respectively (Tables 2 and 3).

**TABLE 2:** Frequency for socio‐demographic characteristics of respondents of KAP study relating to zoonotic diseases.

Variable	Category	Frequency	Valid percentage
Gender	Female	72	59
	Male	51	41
Age	Between 18 and 35	30	24
	Between 36 and 50	54	44
	Greater than 51	39	32
Primary livelihood activity	Farming	69	56
	Others	54	44
Educational status	College	30	24
	High school	48	39
	Elementary school	21	17
	Illiterate	24	19
Respondent status	Farm worker	42	34
	Farm owner	81	66
Residence	Peri‐urban	36	29
	Urban	87	71

**TABLE 3:** Frequency for farm characteristics on KAP study relating to zoonotic diseases.

Variable	Category	Frequency	Valid percentage
Farm size	Medium	48	39
	Small	75	61
Farm type	Intensive	78	63
	Semi‐intensive	45	37
Floor type	Concrete	84	59
	Muddy soil	39	41
Drainage	Have	108	88
	Don't have	15	12
Barn cleaning frequency	Once per day	15	12
	Twice per day	78	63
	Trice per day	19	15
	Above a day	11	9
Calf house	Common	54	44
	Separate	69	56
Major breed	Cross breed	72	58
	Local breed	51	41

#### 3.1.1 Knowledge on Zoonotic Diseases

Out of the 123 respondents, 114(93 %) know about zoonotic disease and 29% of the participants do have training about zoonotic disease. Out of the respondents, 12 (10%) believed that a family or a farm member was sick with disease of animal origin. Majority of respondents know that disease can be transmitted from animals to them through eating raw meat (90%) and drinking raw milk (85%). The proportion of respondents that know assisting animal parturition and collecting aborted foetus and placenta with bare hands expose them to disease were 83% and 80%, respectively. The detailed information used in the building of knowledge score is mentioned in Table 4.

**TABLE 4:** Frequency for knowledge score answers relating to zoonotic diseases.

		Correct response	
Item No.	Knowledge subscale contents	No	%
K1	Do you know about zoonotic disease	114	93
K2	Training about zoonotic disease	36	29
K3	Eating uncooked meat can transmit diseases from animals	111	90
K4	Drinking of raw milk can transmit diseases from animals	105	85
K5	Get infection from environment contaminated from secretions of sick animal	81	66
K6	Disposing aborted foetuses into the environment can spread the diseases	90	73
K7	Contact during animal abortion can cause a public health problem	63	51
K8	Collecting aborted foetuses and placenta with bare hand	99	80
K9	Assisting animals during parturition with bare hand	102	83
K10	Family or farm member sick with a disease of animal origin	12	10
K11	Transmission of diseases through milk or meat	108	88
K12	Mention three diseases that transmitted from animals to humans	54	51

#### 3.1.2 Attitude and Practices on Zoonotic Disease

Among the respondents, 81% believed that zoonotic diseases can be controlled and prevented whereas 80% thought zoonotic diseases do have treatment. The participants who agreed on the risk of raw milk and meat consumption and a fatal risk of milk‐ and meat‐borne diseases were 74% and 76%, respectively. A total of 73% of the respondents understood that assisting animals during delivery and collecting aborted foetuses and placenta with bare hands expose them to disease risks; however, only 17% of them wear protective hand gloves in assisting animal delivery. In addition, the success rates towards prevention of zoonoses through drinking milk after boiling or pasteurization and eating meat after cooking were 61% and 54%, respectively. Detailed information and questions involved in the construction of attitude and practice scores are shown in Table 5.

**TABLE 5:** Frequency table for attitude and practices relating to zoonotic diseases.

		Agree responses		Item		Success response	
Item No.	Attitude subscale contents	No	%	No	Practice subscale content	No	%
A1	Assisting animals during delivery with bare hand exposes you to disease risks	90	73	P1	Drinking of milk after boiling (pasteurization)	75	61
A2	Collecting aborted foetuses & placenta with bare hand exposes you to disease risks	90	73	P2	Eating of meat after Cooking	66	54
A3	Animal health care providers can handle zoonotic disease outbreaks very well	87	71	P3	Separating house of animals	78	63
A4	Animal diseases are dangerous for people	93	76	P4	Assist animal delivery with protected hands	21	17
A5	Zoonotic diseases can be treated	96	78	P5	Washing hands with soap after assisting delivery	120	98
A6	Zoonotic diseases can be prevented	100	81	P6	Avoid any contact with aborted material	78	63
A7	Zoonotic diseases can be controlled	99	80				
A8	Meat‐ and milk‐borne diseases are fatal	93	76				
A9	Risk of disease from drinking raw milk and eating raw meat	91	74				
A10	Only sick cattle could be the source of milk‐ and meat‐borne disease	70	57				

#### 3.1.3 Internal Consistency

Cronbach's α was calculated for subscales. The knowledge and attitude subscales had good internal consistency and reliability with Cronbach's α of 0.711 and 0.756, respectively. The practice subscale had a Cronbach's α of 0.255 which was lower than the minimum acceptable value of 0.7, indicating that this subscale showed inadequate internal consistency and reliability. The overall Cronbach's α (KAP sections) was estimated at 0.831.

#### 3.1.4 Univariable Analyses

Sex, primary livelihood activity and educational status are associated with the zoonotic knowledge score (*p* < 0.05). Concerning attitude score, age, sex, educational status and farm size (*p* < 0.05) were positively associated. In addition, age, educational status, primary livelihood activity, respondent's role, residence, farm type and farm size were associated (*p* < 0.05) with practice score. The detailed results of univariable analyses are displayed in Table 6.

**TABLE 6:** Univariable linear regression analysis, demonstrating the influence of explanatory variables on the outcome variables.

Variable	Category	No_	Knowledge correct response			Attitude agree response			Practice success response		
			Mean	Coffe.	*p*‐value	Mean	Coffe.	*p*‐value	Mean	Coffe.	*p*‐value
	Overall	123	65			74			59		
Sex	Female	72	70	51	0.000	85	14	0.003	62	5	0.162
	Male	51	68			70			67		
Age (Year)	18–35	30	71	6	0.264	80	5	0.471	63	12	0.016
	36–50	54	58			67			55		
	>50	39	77	19	0.000	85	18	0.001	76	20	0.000
P.L.Activity	Farming	69	71	19	0.000	82	9	0.079	62	4	0.240
	Other	54	66			73			67		
Education	Elementary	21	81	29	0.000	80	21	0.002	69	16	0.003
	High school	48	66	14	0.016	81	22	0.004	67	14	0.005
	College	30	76	23	0.000	91	33	0.000	67	13	0.004
	Illiterate	24	52			58			54		
Respondent	Worker	42	66	6	0.093	79	3	0.483	71	12	0.001
	Owner	81	72			76			58		
Resident	Peri‐Urban	36	72	7	0.069	82	9	0.115	58	12	0.004
	Urban	87	65			73			71		
Farm size	Medium	48	72	7	0.062	84	15	0.005	71	12	0.002
	Small	75	65			71			58		
Farm type	Intensive	78	70	3	0.368	80	6	0.241	57	15	0.000
	Semi‐intensive	45	67			74			72		

Abbreviation: P.L.Activity = Primary Livelihood Activity.

#### 3.1.5 Multivariable Analysis

The education level (*p* < 0.05), which was positively associated with the zoonotic disease knowledge score, attitude and practice scores, and the age of farmer (*p* < 0.05), which was negatively associated, were the significant parameters. Regarding attitude score, sex and farm size (*p* < 0.05) were important parameters that were positively correlated. Lastly, the respondent's role, residence of the farm, farm size and its type (*p* < 0.05) were associated with practice scores. The detailed results of multivariable analysis are presented in Table 7.

**TABLE 7:** Multivariable linear regression analysis, demonstrating the influence of explanatory variables on the outcome variables.

Variables	Adjusted *R*2	*p*‐Value
**Knowledge score**		
Age	0.428	0.000
Education		0.000
**Attitude score**		
Sex	0.316	0.003
Age		0.001
Education		0.000
Farm size		0.005
**Practice score**		
Age	0.412	0.000
Education		0.012
Respondent role		0.001
Resident		0.000
Farm type		0.000
Farm size		0.002

#### 3.1.6 Correlation between Knowledge, Attitude and Practice

Through correlation analysis, the Pearson's correlation coefficient (*r*) indicated that there was a moderate positive association between responding correctly in the knowledge section and having the desired attitude (*r*2 = 0.644, *p* = 0.000). There was a positive but weak relationship between correctly responding in the knowledge section and self‐reported good practice (*r*2 = 0.207, *p* = 0.021). Good practices were also positively associated with the desired attitude (*r*2 = 0.329, *p* = 0.000).

## 4 Discussion

Dairy livestock keepers play an important role in the economy of any country, especially developing country like Ethiopia. The economical contribution is not only by generating income to the owners but also by providing employment to rural and peri‐urban community. In this study, the first attempt in Sodo was assessing the KAPs of dairy farm practitioners towards zoonotic diseases. More than half of the participants have sufficient knowledge (65%), positive attitude (74%) and positive practice (59%) towards the transmission, control and prevention of zoonotic diseases. This result indicates that, despite an overall good KAP score about zoonotic diseases, there are some serious knowledge and awareness shortcomings.

Almost all (93%) of the respondents knew about zoonotic disease, so they can be considered as highly knowledgeable about zoonosis. The majority of the respondents knew the transmission of disease from animals to humans by drinking raw milk, eating uncooked meat, assisting animals during parturition and collecting aborted foetuses or placenta with bare hands. The percentage of participants who knew drinking of raw milk is a source of infection was 85%. This figure is higher than a study conducted in Ethiopia's Ada'a district, Oromia, done by Fufa and his colleagues in 2020 (66%) (Abunna, Gebresenbet, and Megersa 2024) and a study conducted in Zimbabwe done by Mosalagae and his colleagues in 2011 (41%) (Mosalagae, Pfukenyi, and Matope 2011) and lower than a study conducted in Ethiopia's North Showa zone by Seyoum and his colleagues in 2016 (99%) (Seyoum et al. 2016), but it is comparable with the finding of Musallan from Jordan conducted in 2015 (87%) (Musallam et al. 2015). In the present study, 90% of the participants knew that eating uncooked meat is a source of infection. In this regard, respondents seem to have a good deal of knowledge concerning the source of disease when eating raw meat. Raw meat consumption has been applied for generations in many social groups located in Russia, Cuba and Africa including Ethiopia (Abera et al. 2016).

Participants who knew that assisting animals during parturition and collecting aborted foetuses or placenta with bare hands as a source of infection were 83% and 80%, respectively. It is much higher than that of Seyoum (Seyoum et al. 2016) where 15% mentioned assisting with cow birth could be a source of infection. The majority of participants believed that zoonotic diseases can be controlled (80%), prevented (81%) and treated (78%), which is much higher than that of Khadayata and Aggarwal (2020) where 33% had knowledge where zoonotic disease can be prevented by maintaining proper hygiene. The indicated prevention methods are using cooked meat, boiling milk, vaccination and separating animals’ house. The participants who agree risk of acquiring disease through the consumption of raw meat and milk were 74%.

This result is much higher than that of Seyoum (Seyoum et al. 2016) where 21% of respondents said raw milk is not more healthy and nutritious than pasteurized or boiled. Acceptable level of attitude was obtained regarding the possibility of contracting zoonotic diseases from only sick animals (57%). That means 42% believed that disease can be acquired from apparently healthy animals much higher than that of Seyoum (Seyoum et al. 2016) who reported 18%. Consumption of meat (90%) is the prime way for zoonosis and milk (85%). Babu (Babu et al. 2015) from India has reported that 22% and 14% of respondents were conscious on the consumption of meat and milk, respectively, causing zoonotic infection. Whereas most livestock keepers were aware of the risk involved through the consumption of animal products, such as milk and meat, as well as direct transmission, for example, by aerosols or direct contact. This good level of awareness helps them likely to prevent from an increased risk of contracting zoonoses, as they are likely to take proper precautions or use protective clothing when dealing with abortions or calves with diarrhoea and during on‐farm activities like milking, cleaning the cowshed or slaughtering cattle. Although livestock keepers might be aware of the risk of consuming raw milk or meat, the habit of consuming raw milk, raw blood or raw or undercooked meat is, however, still common practice, especially among rural communities across the globe and Africa including Ethiopia (Shirima, Kazwala, and Kambarage 2003).

Despite the majority of the participants having sufficient knowledge (65%), positive attitude (74%) and positive practice (59%) in relation to transmission, control and prevention of zoonotic diseases, 42% of them could not reflect their knowledge and attitude towards real practices. The current study is consistent with the finding of the study conducted in Bahir Dar, Ethiopia, having positive prevention practice of 39% (Alemayehu et al. 2024), which is higher than 32% positive practice in a study done by Cakmur in Kars, Turkey (Çakmur et al. 2018). The most important findings are identification of several high‐risk practices: absence of protective equipment while assisting an animal's parturition and handling birth, eating raw meat, drinking raw milk and living in common with animal material, all being universal among the participants. Respondents were highly knowledgeable and approach concerning of the potential health risks of animal zoonosis. Unfortunately, such level of knowledge attitude was not reflected in the real practice where 41% do negative practice such as drinking raw milk, eating raw meat, assisting animal parturition and collecting aborted materials with bare hands. In this study, nearly 61% of dairy farmers mentioned that they consumed milk after boiling; however, 39% of them drink raw milk, which is consistent with Seyoum (Seyoum et al. 2016) who reported 47% practice drinking of raw milk. Similarly, 39% of the respondents have the habit of eating raw meat, which is much higher than 12% of cattle farmers who consume raw meat in Erzurum, Turkey (Özlüa, Ataseverb, and Ataseverb 2020).

Production of animal products, contamination during this production, wrong feeding habits and lack of knowledge can be effective in the transmission of zoonotic diseases (Rajkumar et al. 2016; Tebug 2015). Raw meat consumption creates dangerous situations in terms of public health due to parasite diseases originating from food as well as bacterial diseases; this finding is similar with research works done in most of the African countries (Bintsis 2017; Murrell 2013).

One of the most important findings is the identification of several high‐risk practices, with negligence of protective equipment while assisting an animal's parturition and handling aborted material, being universal among the participants. Only 17% of the participants use protected gloves while assisting cow birth. Similar to this study, workers in Jordan also reported that only 6% of cattle owners wear protective cloths and gloves while dealing with cow birth (Musallam et al. 2015). This low level of awareness may lead to risk practice which most likely exposes them to an increased risk of contracting zoonotic disease such as brucellosis, as they are unlikely to take proper precautions or use protective clothing when dealing with animal birth and abortions. There were differences between the positive knowledge level, attitude and practices of cattle farmers regarding zoonotic diseases and their sex, age, educational level, respondent's role, resident farm type and farm size. Similar reports were done by different researchers across the world. The age and education level have been associated with better zoonotic disease knowledge and practices.

There was a significant difference between education levels and KAPs of zoonotic diseases (*p* < 0.05). It was confirmed that especially the ones who had high school and college education had a knowledge level distinctly higher and their attitude and practices, except for those who were not illiterate, were closely related to each other. In the same manner, studies conducted in Tajikistan, Senegal, Nepal and India reported that livestock farmers with low education level had a low level of KAPs towards protection from zoonotic diseases (Kelly et al. 2018; Lindahl et al. 2015; Prasad et al. 2019; Tebug 2015). In addition to this, the increase in age correlates with good knowledge about zoonosis. These findings can be attributed to the improvement of the education system across the years, the acquaintance of the new generation with technological developments and the introduction of training courses for the education of new farmers in this field.

## 5 Conclusion and Recommendations

This study gives an understanding on the variability of knowledge, attitudes and practices related to zoonotic disease risk among dairy farm communities in Sodo. Generally, an overall good score was observed in knowledge, attitude and practice subscores. However, the observed low level of attitude obtained regarding the possibility of contracting zoonotic diseases from apparently healthy animals, low level of practices in drinking boiled milk and eating cocked meat need urgent intervention. The finding of this study suggested that establishing a desired attitude on the impact of those diseases on public health and their mitigation strategies among the community is vital to reduce the transmission of zoonotic agents from animals to humans. Therefore, awareness should be created about the importance of zoonosis with respect to KAP among livestock keepers by information, education and communication. Community awareness should be included to educate how harmful consumption of raw or unpasteurized milk and uncooked meat and bare hand assistance during parturition and handling of aborted materials. Age and education of the farmer must be in the core of any program oriented towards the improvement of KAPs related to the zoonotic disease. The role of the farmer and the location of the farm determine awareness, disease identification skills, and preventive behavioural practices; thus, it needs attention during community health education program development and awareness should be done to address the above practice gaps to reduce the risk of zoonotic infection to livestock producers and livestock products consumer.

## Author Contributions

**Getachew Derbew Belay**: conceptualization, formal analysis, investigation, methodology, software, writing–original draft. **Amare Bihon Asfaw**: methodology, software, visualization, writing–review and editing. **Hagazi Fantay Tadesse**: data curation, formal analysis, supervision, writing–review and editing. **Asma Seid**: data curation, formal analysis, writing–original draft. All authors have read and agreed to the published version of the manuscript.

## Ethics Statement

Ethical clearance has been obtained from Samara University College of Veterinary Medicine and Animal Science ethical review committee (ERC). The data have been only being used for the intended project and have not been exposed to any third person. The data related to individual identification like names, registration number, mobile number, and others had not been included in the structured questionnaire, so that confidentiality of participants could not be affected.

## Conflicts of Interest

The authors declare no conflicts of interest.

### Peer Review

The peer review history for this article is available at https://publons.com/publon/10.1002/vms3.70197

## Data Availability

The data for this article is fully available and can be provided upon request at any stage of this manuscript processing.
